# Good parenting may not increase reproductive success under environmental extremes

**DOI:** 10.1111/jeb.13358

**Published:** 2018-08-19

**Authors:** Rebecca J. Fox, Megan L. Head, Iain Barber

**Affiliations:** ^1^ School of Life Sciences University of Technology Sydney Ultimo NSW Australia; ^2^ Division of Ecology and Evolution Research School of Biology Australian National University Canberra ACT Australia; ^3^ Department of Neuroscience, Psychology and Behaviour College of Medicine Biological Sciences and Psychology University of Leicester Leicester UK; ^4^ School of Animal, Rural and Environmental Sciences Nottingham Trent University Nottinghamshire UK

**Keywords:** behavioural plasticity, dissolved oxygen, environmental‐dependence, *Gasterosteus aculeatus*, nest, parental care, sexual selection, three‐spined stickleback

## Abstract

For species exhibiting parental care, the way in which parents adjust care behaviour to compensate for environmental change potentially influences offspring survival and, ultimately, population viability. Using the three‐spined stickleback (*Gasterosteus aculeatus*) – a species in which males provide parental care by building and tending a nest and fanning the eggs – we examined how low dissolved oxygen (DO) levels affect paternal care, embryo development and survival. Although levels of nest tending were unaffected by DO level, we found that larger males fanned their embryos more under low oxygen conditions. This resulted in faster rates of embryo development within the clutches of these larger males, but reduced embryo survival at 7 days post‐fertilization compared to clutches of smaller males. Our results suggest that although parents may attempt to compensate for environmental change via alterations to care behaviour, their ability to do so can be dependent on parental phenotype. This sets up the potential for oxygen levels to act on the strength and direction of selection within populations. We discuss possible explanations for the surprising result that supposedly adaptive changes in care behaviour by large males (i.e. increased fanning) led to reduced embryo survival at 7 days post‐fertilization, and whether, as a consequence, acute environmental conditions may have the potential to overwhelm selection on sexual traits.

## Introduction

To maximize reproductive success, many taxa have evolved to provide offspring with parental care (Trivers, 1972; Clutton‐Brock, 1991). Although the type and amount of care that parents provide their offspring vary widely in nature (Gross & Sargent, 1985; Clutton‐Brock, 1991; Cockburn, 2006), the amount of resources that parents invest in offspring generally represents a balance of the costs incurred by the parent (in terms of current energetic expenditure on caring and lost mating opportunities from the enforced asexual period), and the benefits arising from increased offspring survival and/or quality (Trivers, 1972; Maynard Smith, 1977; Gross, 2005).

The balance between these costs and benefits of parental care is likely to depend on the environment in which care takes place, meaning parents should adjust their care behaviour in response to environmental conditions (Carlisle, 1982; Bonsall & Klug, 2011). For instance, in poor nutritional environments the cost for parents of providing care for offspring is greater and so parents often reduce investment in care when nutritional conditions are poor (Carlisle, 1982; Davis *et al*., 1999; Krause *et al*., 2017). Likewise, when environmental conditions are good and therefore likely to support high offspring survival, parents invest more in care (Carlisle, 1982), including investing in weaker offspring signalling the greatest need. For example when resources are plentiful, adult birds will preferentially feed chicks exhibiting the strongest begging levels, despite ignoring such offspring when food is scarce (Davis *et al*., 1999).

In aquatic environments, fish have been shown to adjust their parental care behaviour in response to the amount of dissolved oxygen (DO) in the water. For example, adult male anemonefish (*Amphiprion clarkii* and *A. melanopus)* adjust the amount of time spent tending eggs as ambient DO levels fluctuate over a natural daily cycle, in some cases ceasing fanning activity in response to the lower oxygen requirements associated with reduced levels of egg metabolism at night (Moyer & Bell, 1976; Ross, 1978), and in other cases spending more time tending the eggs during nocturnal hours when DO levels are at a minimum (Green & McCormick, 2005). Likewise, male three‐spined sticklebacks (*Gasterosteus aculeatus*) adjust their care behaviour by engaging in longer bouts of egg fanning during nocturnal periods when DO levels fall (Reebs *et al*., 1984) or reducing fanning when increased turbidity from phytoplankton growth enhances levels of oxygen in the environment (Candolin *et al*., 2008). And, male sand gobies (*Pomatoschistus minutus*) invest greater effort in fanning eggs when they are exposed to low (39%) DO levels (Lissåker *et al*., 2003). This change in behaviour is expected to occur because increased fanning by parents in low oxygen conditions is beneficial to offspring fitness (Green & McCormick, 2005), although it may come at an energetic cost to the caring parent (Jones & Reynolds, 1999a,b).

Most studies that have measured the effects of the environment on parental care have tended to focus on whether care behaviour varies with the environment, and on the costs that these behavioural responses impose on adults (Jones & Reynolds, 1999a,b). In the case of DO content of water, for instance, it is known that oxygen availability is one of the most important factors influencing fish metabolism in early development (Kramer, 1987; Rombough, 1988) and that DO therefore plays a crucial role in offspring development and survival (Cobel, 1961; Herrmann *et al*., 1962; Silver *et al*., 1963; Shumway *et al*., 1964; Garside, 1966). As highlighted above, it is also known that many fish species adjust their parental care in response to DO. Thus, the effects of low DO on offspring may be mitigated by adjustments to the amount and type of care provided by parents, because fanning water over the developing embryos increases water circulation in the nest, removing metabolic products and increasing the availability of oxygen to developing embryos (Wootton, 1976; Green & McCormick, 2005). The effects of environmentally induced changes in parental care behaviour on offspring fitness are less well studied (although see Candolin *et al*., 2008, 2016). This is surprising, because understanding how changes in parental care mediate effects of the environment on offspring is critical to understanding the evolution of parental care behaviour and how organisms might respond to environmental change.

Here, we address this knowledge gap by investigating how male three‐spined sticklebacks adjust their parental care in response to the DO content of the water and look at the consequences of changes in behaviour for both embryo development and survival. We also investigate whether changes in behaviour are related to parental phenotype. Three‐spined sticklebacks are common in coastal marine, brackish and freshwater habitats across the northern hemisphere. In this species, the male undertakes all parental care, with the female playing no role in offspring care after depositing her clutch. Male sticklebacks construct nests, which function in courtship (Kraak *et al*., 1999; Barber *et al*., 2001; Östlund‐Nilsson & Holmlund, 2003; Rushbrook & Barber, 2008; Rushbrook *et al*., 2010) and in protection of eggs and developing embryos (Wootton, 1976). Once a female has spawned in a male's nest, she abandons the eggs and the male enters into a parental care phase, where he cares for eggs by defending the nest against egg predators; fanning water through the nest; and tending the nest (i.e. adjusting nest structure to allow for greater water flow and removing unfertilized, dead and diseased embryos) (Wunder, 1930; van Iersel, 1953; Wootton, 1984). We predicted that males in low DO conditions would spend more time fanning their nests than those in high DO conditions and that males who fanned their nests more under low DO conditions would have faster offspring development and higher offspring survival than males who spent less time fanning. In addition, we predicted that large males would show a higher propensity to alter their care behaviours in response to their environment, by virtue of having more resources at their disposal with which to trade off investment in parental care.

## Materials and methods

### Animals and husbandry

Adult three‐spined sticklebacks were collected from Carsington Reservoir, U.K. (53°3′30″N 1°37′50″W), and held in aquarium facilities at the University of Leicester. Full‐sibling clutches of fertilized eggs were produced via *in vitro* fertilization of these wild‐caught adults and resulting fry were reared to reproductive condition in the laboratory under light and temperature regimes that mimicked those in their natural habitat (described in Head *et al*., 2017). On reaching reproductive maturity (determined by the presence of breeding colouration), fish were separated by sex and maintained in recirculating aquaria in single‐sex groups.

### Experimental design

At the start of the experiment, male sticklebacks that showed nuptial colouration were weighed and measured (wet mass recorded to 0.001 g and standard length recorded to 0.01 mm). They were then transferred to individual aquaria (17.5 × 32 × 17 cm with mixed sand and aquarium gravel substrate) and randomly divided amongst two treatment groups: high DO (maintained at 90–100% air saturation, equivalent to an oxygen level of 8.7–9.7 mg L^−1^ at 16–18 °C) and low DO (maintained at 30–40% air saturation, equivalent to an oxygen level of 2.9–3.9 mg L^−1^ at 16–18 °C). Dissolved oxygen levels in freshwater systems can vary from < 1 to > 20 mg L^−1^ depending on, amongst other things, time of day, season and water depth. Shallow‐water fishes, such as stickleback, tend to require DO levels of between 4 and 14 mg L^−1^, meaning that our high DO level reflected ambient natural conditions and our low DO level reflected the bottom of the range these fish typically inhabit. High and low oxygen treatments were created by bubbling air or nitrogen, respectively, into the water through an air stone. Oxygen levels were monitored twice daily using an oxygen meter (YSI 550A, calibrated daily) and aquaria were held under a 16:8 light:dark photoperiod and temperature of 17 ± 1 °C.

After a 6 h acclimation period, 400 5‐cm‐long black polyester threads were introduced to the aquaria as material for building nests. One day later, and on each subsequent day, each male was presented with a gravid female (housed in a glass jar with mesh top) for a period of 20 min to stimulate nest building. Female presentations continued until a males’ nest was completed. Males were checked daily for the presence of a nest and after 4 days of nest construction, any unused threads were removed from the tank and males were presented with a free‐swimming gravid female of known mass, who had been acclimated to the respective DO treatment for a 2–6‐h period. Prior to introduction of the female, half of the experimental males experienced a reversal in DO conditions and period of acclimation to the new conditions (from high to low DO, or from low to high DO) as part of a separate experiment (see Head *et al*., 2017 for details). However, for each male, courtship, mating, paternal care and associated embryo development all occurred under the same DO treatment level and previous DO conditions experienced were not considered in the analyses presented here due to lack of clear predictions and a desire not to overfit models.

Courtship behaviour was recorded to confirm that spawning had taken place (results presented in Head *et al*., 2017). Four males did not spawn, resulting in sample sizes of 28 males in each of the DO treatments. Females were removed from the aquaria immediately after spawning, to mimic conditions in nature where the female plays no further role in egg or nest tending. Post‐spawning females were then reweighed (wet mass, to 0.001 g) to estimate spawned clutch mass.

### Effect of DO level on male nest tending and egg fanning

Male parental care behaviour was recorded 4 days post‐spawning by an observer seated 2 m from the nesting aquaria using a notebook PC with event recording software (The Observer, Noldus). Each observation lasted 10 min, during which the amount of time spent tending the nest (manipulating nesting material, removing debris from the nest) and fanning the nest (using fins to move water over the clutch, replacing deoxygenated water with oxygenated water) was recorded. We also recorded how many bouts of each behaviour occurred as well as the number of times a male applied glue to the nest and crept through the nest (however, these variables were not analysed due to the infrequency of their occurrence). From these observations, we calculated the proportion of time spent tending the nest and the proportion of time spent fanning the nest. Male parental care behaviours were also recorded at 1 and 7 days post‐spawning, but these data were discarded prior to analyses*,* because other studies show that care behaviours peak at 4 days post‐spawning (Smith & Wootton, 1995; Piike *et al*., 2007). The compactness of each male's nest was also determined from *in situ* photographs taken 4 days post‐spawning using a tripod‐mounted Fuji Finepix s9600 digital camera and calculated as the bulk area of the nest divided by the total area of the nest, following Barber *et al*., 2001 (Fig. S1).

### Effect of male parental care on embryo survival and development

Embryo survival was recorded as the number of eggs in the nest that were still alive after 7 days of incubation (c.f. Candolin, 2000; Pike *et al*., 2007). Estimates of spawned egg mass that had been deposited in the nest were taken (see above), so that we could control for this when looking at embryo survival. Spawned egg mass was calculated as the difference in female mass before and after spawning, following previous studies where change in female mass has been shown to be highly correlated with clutch mass (Kraak *et al*. 1999; Candolin, 2000; Pike *et al*., 2007). Although this is an imperfect measure of the number of eggs deposited in the nest, the need to not disturb nests after eggs were deposited means that it is the best measure we have of initial clutch size. Under our ‘standard’ laboratory conditions, when held at 100% air saturation, eggs from this population normally hatch at 8 days post‐fertilization (V. Macnab, Personal communications).

To quantify embryo development, we defined a scale based on the amount of pigmentation shown by embryos within the egg mass still alive after 7 days of incubation (embryo pigmentation increases with development; see Vrat, 1949; Swarup, 1958). The scoring scale ranged from 1 to 5, where 1 indicated zero to low pigmentation, 2 indicated low to intermediate pigmentation, 3 indicated intermediate pigmentation, 4 indicated intermediate to high pigmentation and 5 indicated high pigmentation (at stage 5, some embryos had also started hatching). These levels of pigmentation correspond to stages 8–12 of embryonic development described in Vrat (1949) and stages 18, 20, 21, 23 and 24 of development described in Swarup (1958). A single value was assigned to each egg mass, based on the level of pigmentation shown by the majority of embryos within that clutch. All clutches were scored by one person (MLH) blind to the treatment and male phenotype/nest attributes that it came from.

### Statistical analysis

To determine the effects of DO on the proportion of time males spent either tending (i.e. time spent tending/trial time) the nest or fanning the nest (i.e. time spent fanning/trial time), we ran two separate generalized linear models (GLM). Proportion of trial time spent engaged in each behaviour was used, because trial time was not always exactly 10 min. The models included DO level as a fixed factor, as well as male body size and nest compactness as covariates. Two‐way interaction terms between DO level and both male body size and nest compactness were included to test predictions that DO level might affect how these attributes influence parental care behaviour. We then ran these models without the interactions to compare the model fit of reduced and full versions. If removal of the interactions did not affect the model fit (assessed using log‐likelihood ratio tests), we interpreted the main effects from the reduced model. Model estimates were obtained using the ‘summary’ function, whereas significance values were obtained using the ‘anova’ function with a type III sums of squares. All males with zero egg survival (a total of three individuals from the low DO treatment and five from the high DO treatment) were excluded from the analyses because we were unable to discern if these clutches had been fertilized and then cannibalized, or never fertilized in the first place. Although filial cannibalism is an interesting and important aspect of male fitness, the absence of eggs in these nests meant that males were not providing parental care. Including observations of their behaviours would therefore have confounded our analyses of environmental effects on parental care. Final sample sizes for these analyses were 18 in the low DO treatment and 21 in the high DO treatment. For the model looking at effects on time spent fanning, residuals of a model where we specified a Gaussian error distribution were not normal (assessed by visualizing histogram of model residuals and conducting a Shapiro–Wilks test) so time spent fanning was power transformed. The appropriate power transformation (2.23) was determined using the powerTransform function in the ‘car’ package of R (Fox & Weisberg, 2011). Once transformed, model residuals met the assumption of normality. For the model looking at time spent tending the nest, we used a quasi‐Poisson error distribution (transformation of the data did not improve the fit of the residuals and a Poisson model was overdispersed).

To determine how offspring fitness was influenced by DO level, we ran two separate analyses. First, to examine the effects of DO level on embryo development (an ordered response variable), we used a cumulative link model (CLM) with a logit link function. In this model, we included DO level as a fixed factor and male body size, nest compactness, initial mass of eggs, the time spent fanning and the time spent tending the nest as covariates. We also included two‐way interactions between DO level and each of the covariates because we predicted that DO level might affect how each one influenced offspring fitness. We then ran this model without the interaction terms and compared the model fit of the reduced and full models using a log‐likelihood ratio test. If removal of the interaction terms did not affect the model fit, we interpreted the main effects from the reduced model. Second, we used a GLM to look at the effects of DO level on embryo survival (the number of live embryos in the clutch 7 days post‐fertilization). This model contained the same terms as the model looking at embryo development and was also run with, and without, two‐way interaction terms to determine whether main effects could be interpreted from the reduced model. We specified a Gaussian error distribution and confirmed residuals of the model met the assumption of normality by visualizing histograms of the model residuals and conducting a Shapiro–Wilks test. As with previous models, all males with zero egg survival were excluded from these analyses because we were unable to discern if these clutches had been fertilized or not. All analyses were conducted in R version 3.2.0, with GLMs run using the ‘lme4’ package (Bates *et al*., 2015) and CLM run using the ‘ordinal’ package (Christensen, 2015).

All experiments were carried out in the conditions stated, and all measures taken during the experiment and all data exclusions have been reported.

## Results

### Effect of dissolved oxygen level on male parental care

**Figure 1:**
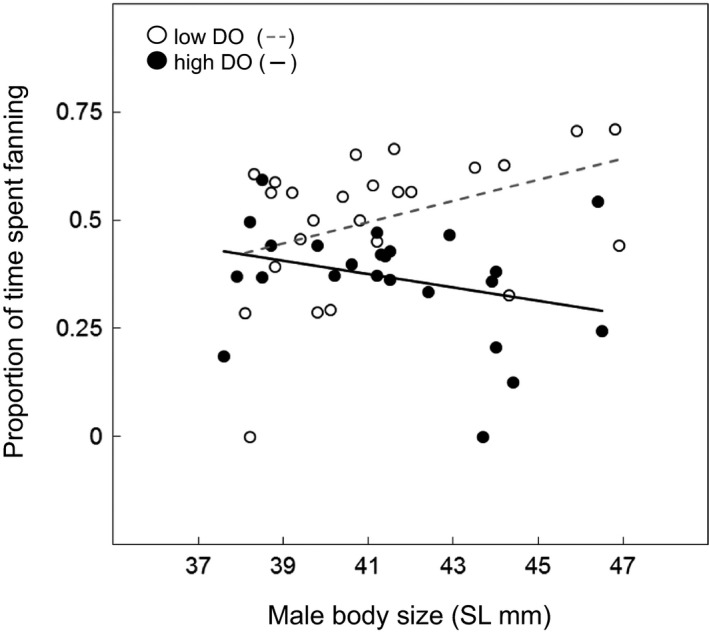
Effect of dissolved oxygen (DO) level and body size (standard length) on the proportion of observation period spent fanning eggs by male three‐spined sticklebacks (*Gasterosteus aculeatus*). Low DO treatment individuals are represented by open circles (line of best fit ‐ ‐), and high DO treatment individuals are represented by black circles (line of best fit —).

The proportion of time males spent tending their nest during the parental care observations was not affected by DO level (*χ* ^2^ = 0.318, *P *=* *0.573), male size (*χ* ^2^ = 0.029, *P *=* *0.864), nest compactness (*χ* ^2^ = 0.458, *P *=* *0.481) or the interactions between the terms (i.e. removal of interaction terms did not alter the fit of the model (*F*_(2,39)_ = 0.393, *P *=* *0.678, Table S1)). However, the proportion of time males spent fanning their nest during parental care observations showed a significant interaction between DO level and male size (*χ* ^2^ = 6.896, *P *=* *0.009) and removing the interaction terms significantly influenced the fit of the model (*F*_(2,39)_ = 4.401, *P *=* *0.019, Table S2). Under high DO conditions, larger males fanned less than smaller males, whereas under low DO conditions, larger males fanned their nests more than smaller males (Fig. 1).

### Effect of dissolved oxygen level on embryo development and survival

**Figure 2:**
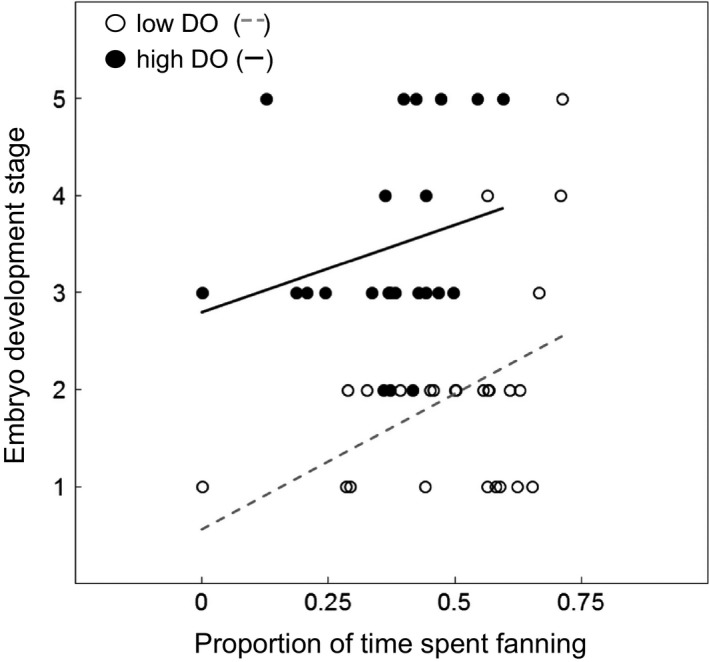
Effect of dissolved oxygen level and proportion of observation period spent fanning eggs on embryo development in the three‐spined stickleback (*Gasterosteus aculeatus*). Embryo development (*y*‐axis) is on an ordinal scale of level of embryo pigmentation that relates to key developmental stages outlined in Vrat (1949) (see Methods). Low DO treatment individuals are represented by open circles (line of best fit ‐ ‐ ‐), and high DO treatment individuals are represented by black circles (line of best fit —).

Embryo development was significantly influenced by both DO level (*z *=* *−4.332, *P *<* *0.001) and the proportion of time males spent fanning their nest (*z *=* *2.110, *P *=* *0.035). Embryos developing in the low DO treatment were less developed at 7 days post‐spawning than embryos experiencing high DO, and males that spent more time fanning had embryos that were more developed (Fig. 2). Neither male body size (*z *=* *1.242, *P *=* *0.214), nest compactness (*z *=* *−0.654, *P *=* *0.513) nor the proportion of time males spent tending the nest (*z *=* *−1.183, *P *=* *0.237) influenced embryo development, and removing the interactions did not alter the fit of the model (*χ* ^2^ = 2.813, *P *=* *0.589, Table S3).

**Figure 3:**
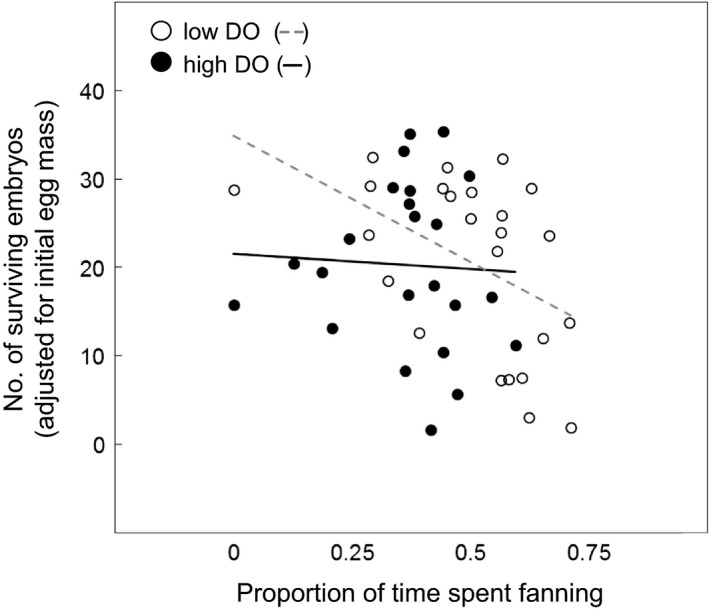
Effect of dissolved oxygen level and proportion of observation period spent fanning on embryo survival (# eggs at 7 days post‐fertilization*initial egg mass^−1^) in the three‐spined stickleback (*Gasterosteus aculeatus*). Low DO treatment individuals are represented by open circles (line of best fit ‐ ‐ ‐), and high DO treatment individuals are represented by black circles (line of best fit —).

Embryo survival was significantly influenced by the interaction between the proportion of time males spent fanning the nest and DO level (*χ* ^2^ = 5.916, *P *=* *0.015). Under high DO conditions, embryo survival was similar across all levels of fanning, whereas under low DO conditions, embryo survival was negatively related to level of fanning effort, meaning that males who invested more in fanning experienced lower levels of embryo survival (Fig. 3). Removal of all interactions from the model did not influence the model fit (*F*_(4,35)_ = 2.542, *P* = 0.059, Table S4) and so we interpret the main effects from a reduced model. The initial mass of eggs spawned in the nest was strongly and positively related to embryo survival (*χ* ^2^ = 17.869, *P *<* *0.0001). However, embryo survival was independent of male body size (*χ* ^2^ = 0.987, *P *=* *0.321), nest compactness (*χ* ^2^ = 0.824, *P *=* *0.364) and the amount of time males spent tending the nest (*χ* ^2^ = 0.008, *P *=* *0.928).

## Discussion

Our study provides evidence of environmentally dependent parental care in the three‐spined stickleback under conditions of oxygen depletion. Although males caring for offspring in low DO conditions did not spend a greater proportion of time tending their nest, they did spend a greater proportion of their time fanning eggs. Further, we found that how males adjust fanning behaviour in response to DO levels was dependent on their size. In low DO conditions, larger males tended to spend more time fanning eggs than smaller males, whereas in high DO conditions, larger males tended to spend less time fanning. Although our sample sizes are small, and so our results should be interpreted with caution, taken at face value, this result could suggest that low oxygen environments act to increase the strength of selection for large males, which have previously been shown to be more attractive to females (Head *et al*., 2017). However, this change in parental care behaviour did not translate to higher embryo survival. In fact, large males who spent more time fanning under the persistent low DO conditions used in this study actually experienced lower levels of embryo survival at 7 days post‐fertilization than males who spent less time fanning (which were typically smaller individuals). Although we do not have data on the final hatching success of embryos (due to the need to destructively sample clutches to measure survival and development at 7 days post‐fertilization), at the scale tested here our study suggests there may be the potential for acute periods of extreme low oxygen to dampen current levels of selection for large males arising through female mate choice. More studies based on larger sample sizes could be helpful in determining the repeatability and reliability of our finding that acute periods of low DO could impact on the strength of selection within particular populations.

We found that large males increased the proportion of time spent fanning when caring for young under low oxygen conditions, but small males did not. Interestingly, the reverse was the case under high oxygen conditions, with large males spending a lower proportion of time fanning eggs than small males. Increasing fanning in response to low oxygen levels is generally viewed as an adaptive behavioural response in fishes that provide parental care (Reebs *et al*., 1984; Hale *et al*., 2003; Lissåker *et al*., 2003; Pike *et al*., 2007; : Candolin *et al*., 2008). For example, under low oxygen conditions male gobies increase both the time spent fanning their eggs and the tempo of fanning to achieve hatching success rates equivalent to those of males caring for clutches under control conditions (Jones & Reynolds, 1999a). However, increased fanning is energetically demanding for males (Kramer, 1987; Jones & Reynolds, 1999a), particularly in oxygen‐depleted environments. If large males are better able to meet the energetic demands of increased fanning behaviour in low oxygen conditions, this could explain why, of the males assigned to the low DO treatment in the current study, only larger individuals exhibited a shift in behaviour towards increased fanning activity. Although our results are based on relatively small sample sizes and so will require further verification, they do highlight the potential for environmentally induced changes in parental care behaviour to depend on parent phenotype (see also Pike *et al*., 2007). Such patterns could have broad implications for how animals adapt to and persist in poor environments due to the altered relationship between traits favoured by sexual selection and those favoured in parental care impacting on the strength and direction of selection (Candolin *et al*., 2007). Future studies investigating how environmental change affects the viability of species in which parents provide parental care would benefit by considering how the environment influences the relationship between sexual selected traits and parenting success.

In common with previous studies, we found that embryos in the low DO treatment had slower development rates than those in high DO (Reebs *et al*., 1984; Lissåker *et al*., 2003; Green & McCormick, 2005). In addition, we found that, under conditions of low DO, increased investment in time spent fanning by larger males increased embryo development rates. Given the documented role of oxygen as a critical factor in embryo development (Kramer, 1987; Rombough, 1988) and the fact that increased fanning is known to speed up development in both high and low oxygen conditions (Silver *et al*., 1963; Shumway *et al*., 1964; Garside, 1966), this result was expected. However, more surprisingly we found that, under low oxygen conditions, males who spent a greater proportion of their time fanning eggs – typically, the larger individuals – experienced lower levels of embryo survival as measured at 7 days post‐fertilization than males who spent less time fanning. If this result were to be indicative of embryo survival to hatching, then low DO conditions could effectively overwhelm or eliminate selection arising from natural female preferences for larger males. A priority of further studies should be to record embryo survival to hatching to examine whether or not this could be the case.

Our finding that large males who increase their fanning effort under low DO conditions actually have lower rates of embryo survival at the 7 days post‐fertilization point is likely to be viewed as counterintuitive and we therefore offer some potential explanations. First, the result may arise because of the persistent nature of low oxygen conditions in our experiment, which may ultimately constrain a male's ability to compensate for the poor environment. At the DO level used in this study (2.9–3.9 mg L^−1^ at 16–18 °C), males with high rates of fanning may simply not have been able to deliver the oxygen levels required by embryos as they reached the later stages of development (as oxygen requirements are positively correlated with embryo development: Reebs *et al*., 1984; Giesing *et al*., 2011). In this case, the investment in fanning by males under low DO conditions may have merely served to speed the rate at which embryos reached the developmental stage at which oxygen becomes a limiting factor, ultimately resulting in a stalling of development and death. If this is the case, then smaller males exhibiting less fanning may have eventually recorded similar, or potentially higher, levels of embryo mortality under low DO conditions as their more slowly developing embryos reached the critical stage of development.

Although we believe the above explanation for lower embryo survival with increased fanning in low oxygen conditions is the most parsimonious, there are alternatives worth discussing. One, is that despite costs associated with decreased survival of embryos, that increased fanning provides an overall benefit by speeding up offspring development because it decreases the time that offspring spend at vulnerable life stages when oxygen levels are low (Klug & Bonsall, 2014). For example, if offspring have a higher risk of mortality when in the egg than as free‐swimming fry when oxygen levels are low, then speeding up egg development could still be better than the alternative of spending longer in the nest under such conditions. Finally, we offer the suggestion that if fanning behaviour is a highly heritable (> 0.9) trait in the three‐spined stickleback (as proposed by Bell *et al*., 2018), then the behaviour of larger males, whereas adaptive under DO levels that more typically characterize the environment of this particular population (4–14 mg L^−1^), may be maladaptive under the acute low DO conditions of our study for reasons discussed above. In our view, however, the difficulty of teasing apart the effects of genes and environment in transmission of behavioural traits makes this a much more tenuous explanation. Further studies that look at hatching success and offspring performance once fry leave the nest are now required to determine whether changes in male parental care behaviour exhibited by large males in low oxygen conditions are adaptive.

Adaptive explanations for the variety of parental care behaviour observed between species and populations are now generally well accepted (Gross & Sargent, 1985; Winkler, 1987; Kokko & Jennions, 2008; Klug & Bonsall, 2010). That is, parents provide care in a way that balances the costs to themselves and the benefits for their offspring within the environmental parameters under which those behaviours have evolved. However, human‐induced environmental change is driving alterations to ecosystems at an unprecedented rate and scale. For species whose life history strategy encompasses obligate parental care, how parents alter their care behaviour in response to this rapid environmental change, and how this in turn influences offspring viability, selection and population persistence, is less well understood (Lindström, 1999). Key questions include – do parents adjust or modify their behaviour to compensate for the effects of environmental change? do such modifications improve reproductive output? and does this have consequences for population viability? If the answers to these questions are ‘yes’, the ability of parents to adjust care behaviour could prove an important pathway via which species with parental care might adapt to environmental change. There is theoretical (Schultz, 1991; Bonsall & Klug, 2011) and empirical (Westneat *et al*., 2013) work that now suggests the ability of parents to adjust care behaviour in response to environmental stochasticity will play a crucial role in these species’ ability to adapt to environmental change. Although many studies have shown that parents can adjust their behaviour in response to the environment (e.g. see review of how parents adjust nesting behaviour Mainwaring *et al*., 2017), our study, if repeatable, may call into question whether such adjustments necessarily compensate for poor rearing environments. We stress the importance of conducting further empirical studies examining the fitness implications of behavioural adjustments to parenting in different environments to determine whether and how species will be able to cope with rapid environmental change.

## Supporting information


**Figure S1** Illustration of methodology used to calculate the “compactness” of nests constructed by male three‐spined sticklebacks (following Barber *et al*., 2001).
**Table S1** Effect of DO treatment on the amount of time males spent tending their nest: Model outputs from full (including interaction terms) and reduced (main effects) models described in Materials and Methods and statistical comparison of model fits.
**Table S2** Effect of DO treatment on the amount of time males spent fanning their nest: Model outputs from full (including interaction terms) and reduced (main effects) models described in Materials and Methods and statistical comparison of model fits.
**Table S3** Effect of DO treatment on embryo development: Model outputs from full (including interaction terms) and reduced (main effects) models described in Materials and Methods and statistical comparison of model fits.
**Table S4** Effect of DO treatment on embryo survival: Model outputs from full (including interaction terms) and reduced (main effects) models described in Materials and Methods and statistical comparison of model fits.
