# Supplementary material for: Good parenting may not increase reproductive success under environmental extremes
Source: J Evol Biol. 2018 Aug 19;31(11):1638–46. doi: 10.1111/jeb.13358 (PMC6849592; doi:10.1111/jeb.13358)
Supplement: Supplementary file 1 — Figure S1 Illustration of methodology used to calculate the “compactness” of nests constructed by male three‐spined sticklebacks (following Barber et al., 2001). Table S1 Effect of DO treatment on the amount of time males spent tending their nest: Model outputs from full (including interaction terms) and reduced (main effects) models described in Materials and Methods and statistical comparison of model fits. Table S2 Effect of DO treatment on the amount of time males spent fanning their nest: Model outputs from full (including interaction terms) and reduced (main effects) models described in Materials and Methods and statistical comparison of model fits. Table S3 Effect of DO treatment on embryo development: Model outputs from full (including interaction terms) and reduced (main effects) models described in Materials and Methods and statistical comparison of model fits. Table S4 Effect of DO treatment on embryo survival: Model outputs from full (including interaction terms) and reduced (main effects) models described in Materials and Methods and statistical comparison of model fits. [file JEB-31-1638-s001.docx]

**Supporting Information**

**Good parenting by attractive males may not increase reproductive success under environmental extremes**

**
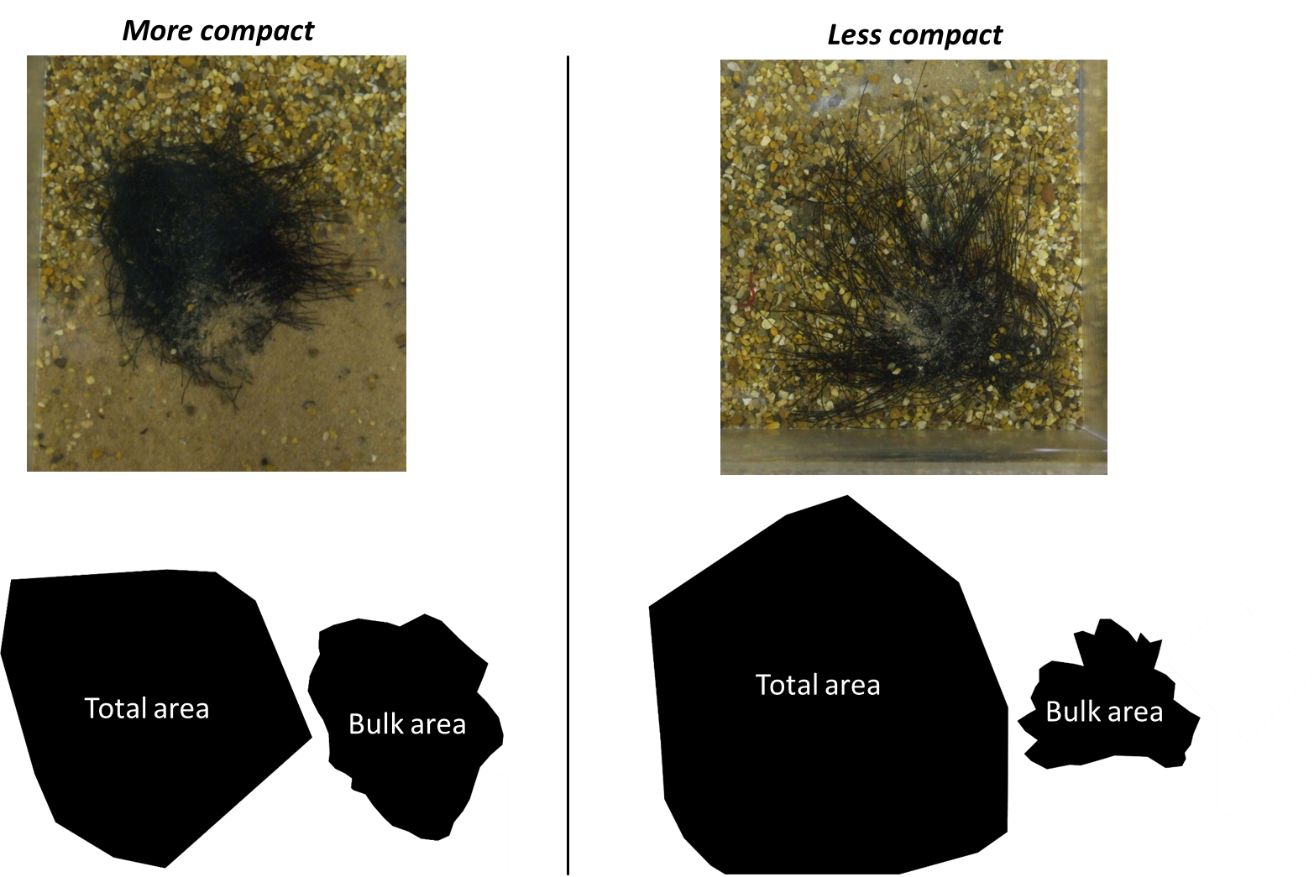
**

**Figure S1:** Illustration of methodology used to calculate the “compactness” of nests constructed by male three-spined sticklebacks (following Barber et al. 2001). Total area is calculated from the minimum convex polygon enclosing all visible nest material. Bulk area is the area covered by the portion of the nest through which no substratum is visible. Nest compactness is then calculated as (bulk area. total area ^-1^).

**Table S1.** Effect of DO treatment on the amount of time males spent tending their nest: Model outputs from full (including interaction terms) and reduced (main effects) models described in Materials and Methods and statistical comparison of model fits.

|  | Estimate | | Std. Error | χ^2^ | p |
| --- | --- | --- | --- | --- | --- |
| **Full model** |  | |  |  |  |
| (Intercept) | 1.439 | | 3.213 |  |  |
| DO Level | 2.708 | | 4.857 | 0.318 | 0.573 |
| Male_size | 0.014 | | 0.081 | 0.029 | 0.864 |
| Nest_comp | 0.680 | | 0.966 | 0.458 | 0.481 |
| DOL*Malesize | -0.061 | | 0.122 | 0.253 | 0.615 |
| DOL*Nestcomp | -1.092 | | 2.072 | 0.284 | 0.594 |
|  |  | |  |  |  |
| **Reduced model** |  | |  |  |  |
| (Intercept) | 2.625 | | 2.334 |  |  |
| DO Level | -0.172 | | 0.297 | 0.335 | 0.563 |
| Male_size | -0.013 | | 0.058 | 0.049 | 0.825 |
| Nest_comp | 0.519 | | 0.828 | 0.388 | 0.533 |
|  |  | |  |  |  |
| **Comparison of fit** |  | |  |  |  |
| Residual (df) | | Deviance (df) | | F | P |
| full 313.16 (37) | |  | |  |  |
| reduced 319.60 (39) | | -6.440 | | 0.393 | 0.678 |

No significant effect of removing interaction terms, so main effect reported from reduced model

**Table S2.** Effect of DO treatment on the amount of time males spent fanning their nest: Model outputs from full (including interaction terms) and reduced (main effects) models described in Materials and Methods and statistical comparison of model fits. Significance values obtained using the “Anova” function with a type III sums of squares.

|  | Estimate | | Std. Error | χ^2^ | p |
| --- | --- | --- | --- | --- | --- |
| **Full model** |  | |  |  |  |
| (Intercept) | 5.880 | | 2.357 |  |  |
| DO Level | -8.199 | | 3.281 | 6.245 | 0.012 |
| Male_size | -0.086 | | 0.059 | 2.174 | 0.140 |
| Nest_comp | 0.477 | | 0.737 | 0.419 | 0.517 |
| DOL*Malesize | 0.214 | | 0.081 | 6.896 | **0.009** |
| DOL*Nestcomp | 0.862 | | 1.345 | 0.412 | 0.522 |
|  |  | |  |  |  |
| **Reduced model** |  | |  |  |  |
| (Intercept) | 1.238 | | 1.779 |  |  |
| DO Level | 0.941 | | 0.227 | 17.214 | <0.001 |
| Male_size | 0.025 | | 0.044 | 0.316 | 0.574 |
| Nest_comp | 0.509 | | 0.663 | 0.591 | 0.442 |
|  |  | |  |  |  |
| **Comparison of fit** |  | |  |  |  |
| Residual (df) | | Deviance | | F | p |
| full 16.155 (37) | |  | |  |  |
| reduced 19.998 (39) | | -3.843 | | 4.401 | **0.019** |

Significant effect of removing interaction terms, so full model reported

**Table S3.** Effect of DO treatment on embryo development: Model outputs from full (including interaction terms) and reduced (main effects) models described in Materials and Methods and statistical comparison of model fits.

|  | Estimate | | Std. Error | z | p |
| --- | --- | --- | --- | --- | --- |
| **Full model** |  | |  |  |  |
| DO Level | -5.668 | | 10.521 | -0.539 | 0.590 |
| Male_size | 0.088 | | 0.170 | 0.515 | 0.606 |
| Nest_comp | -0.525 | | 2.096 | -0.251 | 0.802 |
| Time fanning | 2.798 | | 2.981 | 0.939 | 0.348 |
| Time tending | -3.215 | | 4.062 | -0.791 | 0.429 |
| DOL*Malesize | -0.002 | | 0.264 | -0.008 | 0.994 |
| DOL*Nestcomp | -3.663 | | 4.069 | -0.900 | 0.368 |
| DOL*fanning | 7.147 | | 5.339 | 1.339 | 0.181 |
| DOL*tending | -6.997 | | 8.071 | -0.867 | 0.386 |
|  |  | |  |  |  |
| **Reduced model** |  | |  |  |  |
| DO Level | -3.917 | | 0.904 | -4.332 | **<0.001** |
| Male_size | 0.160 | | 0.129 | 1.242 | 0.214 |
| Nest_comp | -1.157 | | 1.769 | -0.654 | 0.513 |
| Time fanning | 4.793 | | 2.272 | 2.110 | **0.035** |
| Time tending | -4.133 | | 3.493 | -1.183 | 0.237 |
|  |  | |  |  |  |
| **Comparison of fit** |  | |  |  |  |
| AIC (# par) | | logLik | | χ^2^ | P |
| full 127.92 (13) | | -50.960  -52.367 | |  |  |
| reduced 122.73 (9) | |  |  | 2.813 | 0.589 |

No significant effect of removing interaction terms, so main effects reported from reduced model

**Table S4.** Effect of DO treatment on embryo survival: Model outputs from full (including interaction terms) and reduced (main effects) models described in Materials and Methods and statistical comparison of model fits.

|  | Estimate | | Std. Error | χ^2^ | p |
| --- | --- | --- | --- | --- | --- |
| **Full model** |  | |  |  |  |
| (Intercept) | 56.860 | | 86.501 |  |  |
| DO Level | -78.137 | | 118.837 | 0.432 | 0.511 |
| Male_size | -2.049 | | 2.067 | 0.982 | 0.322 |
| Nest_comp | 1.833 | | 25.217 | 0.005 | 0.942 |
| Time fanning | -1.225 | | 36.241 | 0.001 | 0.973 |
| Time tending | 68.924 | | 52.115 | 1.749 | 0.186 |
| Initial egg mass | 304.128 | | 66.152 | 21.136 | <0.001 |
| DOL*Malesize | 3.468 | | 3.010 | 1.328 | 0.249 |
| DOL*Nestcomp | 53.729 | | 49.083 | 1.198 | 0.274 |
| DOL*fanning | -139.494 | | 57.351 | 5.916 | **0.015** |
| DOL*tending | -151.679 | | 97.508 | 2.419 | 0.119 |
|  |  | |  |  |  |
| **Reduced model** |  | |  |  |  |
| (Intercept) | 58.608 | | 59.287 |  |  |
| DO Level | 5.853 | | 8.908 | 0.432 | 0.511 |
| Male_size | -1.426 | | 1.436 | 0.987 | 0.321 |
| Nest_comp | 20.410 | | 22.491 | 0.824 | 0.364 |
| fanning | -44.721 | | 27.802 | 2.588 | 0.108 |
| tending | -3.946 | | 43.531 | 0.008 | 0.928 |
| Initial egg mass | 247.310 | | 58.505 | 17.869 | **<0.001** |
|  |  | |  |  |  |
| **Comparison of fit** |  | |  |  |  |
| Residual (df) | | Deviance | | F | p |
| full 14328 (31) | | -4698.9 | |  |  |
| reduced 19027 (35) | |  |  | 2.542 | 0.059 |

No significant effect of removing interaction terms, so main effect reported from reduced model
